# Diagnosis of Non-ST-Elevation Acute Coronary Syndrome by the Measurement of Heart-Type Fatty Acid Binding Protein in Serum: A Prospective Case Control Study

**DOI:** 10.1155/2014/624930

**Published:** 2014-02-05

**Authors:** Priscilla Abraham Chandran, Basharat Ara Wani, Oruganti Sai Satish, Noorjahan Mohammed

**Affiliations:** ^1^Department of Biochemistry, Nizam's Institute of Medical Sciences, Punjagutta, Hyderabad, Andhra Pradesh 500082, India; ^2^Department of Cardiology, Nizam's Institute of Medical Sciences, Punjagutta, Hyderabad, Andhra Pradesh 500082, India

## Abstract

A prospective case control study was undertaken to evaluate the diagnostic performance of serum heart-type fatty acid binding protein (HFABP) in comparison to cardiac TnT and TnI in 33 patients admitted with chest pain, diagnosed as NSTE-ACS (non ST elevation acute coronary syndrome) and 22 healthy controls. Area under the receiver operating curve (AUC) was highest for H-FABP (AUC 0.79; 95% CI 0.66–0.89) versus cTnI (AUC 0.73; 95% CI 0.59–0.84) and cTnT (AUC 0.71; 95% CI 0.57–0.83). The H-FABP level above 6.5 ng/mL showed 56.7% (CI 37.4–74.5) sensitivity, 0.5 (95% CI 0.3–0.7) negative likelihood ratio (−LR), 100% (CI 84.6–100.0) specificity, and 100% (CI 79.4–100.0) positive predictive value (PPV), 62.9% (CI 44.9–78.5) negative predictive value (NPV). cTnI level above 0.009 *μ*g/L had 40% (CI 22.7–59.4) sensitivity, 0.6 (95% CI 0.4–0.8) −LR, 100% (CI 84.6–100.0) specificity, 100% (CI 73.5–100.0) PPV, and 55% (CI 38.5–70.7) NPV. cTnT showed 46.7% (CI 28.3–65.7) sensitivity, 0.5 (95% CI 0.4–0.7) −LR, 100% (CI 84.6–100.0) specificity, 100% (CI 76.8–100.0) PPV, and 57.9% (CI 40.8–73.7) NPV at level above 9 *μ*g/L. +LR were 12.5 (95% CI 1.8–86.8), 1.7 (95% CI 1.0–3.0), and 1.2 (95% CI 0.8–1.9) for H-FABP, cTnI, and cTnT respectively. In conclusion measurement of H-FABP is a valuable tool in the early diagnosis of patients with chest pain (6–8 hrs) and seems to be a preferred biomarker in the differential diagnosis of NSTE-ACS. More studies are needed to determine whether serum H-FABP further improves diagnostic performance.

## 1. Introduction

The criteria for the diagnosis of myocardial infarction have been redefined recently, as reported in a consensus document of the European Society of Cardiology (ESC) and the American College of Cardiology (ACC) [1]. The increased risk associated with even minor amounts of myonecrosis has led to the concept that any amount of myocardial necrosis should be defined as a myocardial infarction [1]. This change in perspective will lead to an increase in the number of cases of AMI that are recognized (improved sensitivity). Presumably, fewer false-positive diagnoses will occur (improved specificity) owing to the improved performance of newer diagnostic technologies. In contrast with the older World Health Organization criteria, the ESC/ACC criteria place a much greater emphasis on the role of biochemical cardiac markers in the diagnosis of AMI. However, the selection of the most optimal cardiac marker (or combination of markers) remains controversial [2]. H-FABP also elevated in heart failure and unstable angina patients (UAP) [3]. The distinction between unstable angina and non-ST-segment elevation is based on the absence (unstable angina) or presence (non-ST-segment elevation myocardial infarction) of an elevated cardiac marker detectable in appropriately timed blood specimens [4]. Although no marker meets all of the desired features of a biomarker, the more specific cardiac markers, troponin T (cTnT) and troponin I (cTnI), have a number of attractive characteristics. In addition to being useful for diagnosis, the troponins also permit the estimation of prognosis and risk stratification of patients with ACS. Cardiac TnT (cTnT) and cardiac TnI (cTnI), therefore, have been accepted as the “gold standard” markers in the evaluation of patients with ACS [5], but they have several limitations. Though very specific to cardiomyocytes necrosis, they are unable to differentiate ischemia from other mechanisms of injury [6] and cTn release is often not detectable until 6–9 hours following injury, delaying diagnosis. Troponin levels are also reported to be elevated in patients with other medical conditions, such as congestive heart failure, myocarditis, or renal failure, making the test less suitable for diagnosis of ACS. These limitations have prompted us to evaluate the diagnostic properties of Heart-Type Fatty Acid Binding Protein in serum (H-FABP) [7]. It is a cardiomyocyte cytosolic protein and is small and quickly released into the circulation in myocardial injury. H-FABP levels are detectable in blood 2-3 hours following initial injury, and they return to normal within 12–24 hours [8]. Due to low normal levels in serum and high tissue content, a rapid rise above clinical cut-off level is warranted [7]. It is approximately 20-fold more specific than myoglobin and more sensitive than cTn for cardiac muscle [8] in the early hours of chest pain. Therefore, H-FABP has more potential value in early detection of ACS [9]. We studied the diagnostic test properties of serum H-FABP as compared to cardiac TnT and TnI in patients with chest pain admitted in ICCU in a tertiary care hospital.

## 2. Subject and Methods

This is a case control study of 30 patients and 22 controls between December 2011 and May 2012 at the Department of Biochemistry and Department of Cardiology, Nizam's Institute of Medical Sciences, Hyderabad, A.P, India. Patients aged between 30 and 60 yrs. with first episode of chest pain within 6–8 hours were enrolled. All these patients were admitted to ICCU and diagnosed as UA or Non-ST-elevation acute coronary syndrome based on clinical and 12 lead ECG. The protocol of ESC was followed for the diagnosis of NSTE-ACS. Patients without chest pain but with other symptoms suggestive of an atypical presentation of ACS by the assessing physician in the emergency department were also included. The control group had 22, age and sex matched apparently healthy voluntary blood donors. Patients with chest pain more than 8 hours duration, noncardiac chest pain, recent injuries, and renal failure were excluded.

Informed written consent was obtained from all the subjects and the study was approved by the Institutional Ethics Committee.

### 2.1. Clinical Assessment

All patients underwent an initial clinical assessment that included a clinical history, physical examination, 12-lead electrocardiogram (ECG), continuous ECG monitoring, standard blood pressure measurements, and chest radiography in the ICCU. ECG and systolic and diastolic blood pressures (measured in the supine position) were assessed under standardized conditions. Information with respect to smoking status (smoker versus nonsmoker), alcoholism, and preexisting hypertension was obtained.

### 2.2. Laboratory Analysis

5 mL venous blood was drawn in plain tubes from patients within eight hours of symptom onset; serum was separated within half an hour and stored at −70°C until analysis and the samples were thawed only once. The samples were processed for H-FABP by quantitative immunoturbidimetric method (Randox Laboratories, Ltd., Co., Antrim, United Kingdom) and cardiac troponin T and troponin I by time resolved immunofluorescence method (AQT90 FLEX, Radiometer, Denmark).

### 2.3. Statistical Analysis

Sample size was calculated prospectively on the basis of obtaining estimates of sensitivity and specificity with adequate precision. Statistical software, MedCalc Statistical Software version 12.7.7 (MedCalc Software bvba, Ostend, Belgium; http://www.medcalc.org/; 2013) was used for all data analysis. Descriptive statistics of normal data is reported as mean and SD and that of nonnormal data is reported by using five-numbered summary consisting of minimum; 25th, 50th, and 75th percentile; and maximum [10]. Students *t*-test was used to test the significance of the difference between the means of normally distributed data and the Mann-Whitney *U* test was used to compare biomarker levels (nonnormally distributed data) between two independent groups (e.g., patients diagnosed as NSTE-ACS and control subjects). Receiver operating characteristic (ROC) curves were generated for each biomarker to assess their performance and were analyzed for the standard measures of test validity including sensitivity, specificity, predictive values, and likelihood ratios with 95% confidence intervals. The comparison of areas under the ROC curves (AUC) was performed. The optimal cut-off point for dichotomizing serum concentrations of H-FABP was selected to maximize the Youden index [11]. The receiver operating characteristics curves were calculated to show the variability of sensitivity and specificity for cut-off points of different concentrations of H-FABP, cTnT, and cTnI which were measured as continuous variables (see Table 3). All hypothesis testing was 2-tailed and *P* < 0.05 was considered statistically significant.

## 3. Results 

### 3.1. Baseline Characteristics

The study population demographics of 30 patients, 20 male and 10 female, are shown in Table 1. Those admitted with an AMI were older and had risk factors like smoking, alcohol, hypertension, and diabetes. None of them had renal impairment. There was no significant difference between the mean levels of total cholesterol (TC), low density lipoprotein cholesterol (LDL-C), very low density lipoprotein cholesterol (VLDL-C), triglycerides (TG), urea, and creatinine. Significant difference was noted for serum high density lipoprotein cholesterol (HDL-C), TC/HDL-C between cases, and controls.

None of the biomarkers for cases were normally distributed in the study.  Table 2 compares the median, minimum, and maximum of each biomarker (and the IQR) in the patients. Median levels of cTnI, cTnT, and H-FABP, (*P* values: <0.001, <0.0004, and <0.0001, resp.) are all significantly higher in patients when compared with the controls.

**Figure 1:**
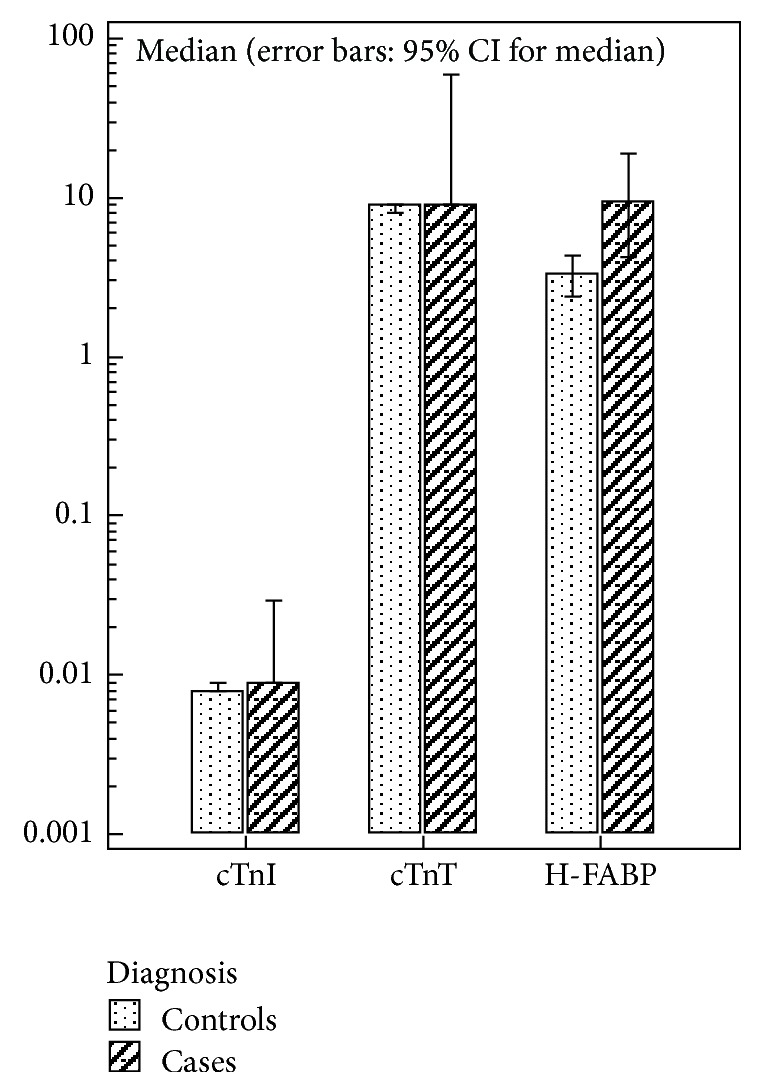
Clustered multiple variables graph.

All the three levels are log transformed and presented as box and whiskers plots (25th percentile, median, 75th percentile) and error bar for 95% CI graph comparing subgroups across different biomarkers in Figure 1.

### 3.2. Mann-Whitney *U* Test (Independent Samples)

The significance of the difference between the cardiac biomarkers was investigated using Mann-Whitney *U* test. The serum concentration levels of cTnT and cTnI in cases were significantly different from that of the levels in the controls, Mann-Whitney *U* = 192, *P* = 0.008 and *U* = 180, *P* = 0.003, respectively. The serum concentration levels of H-FABP in cases were significantly different from that of the levels in the controls, Mann-Whitney *U* = 138, *P* = 0.0004. Though all the three biomarkers were significantly different between cases and controls, H-FABP was highly significant.

**Figure 2:**
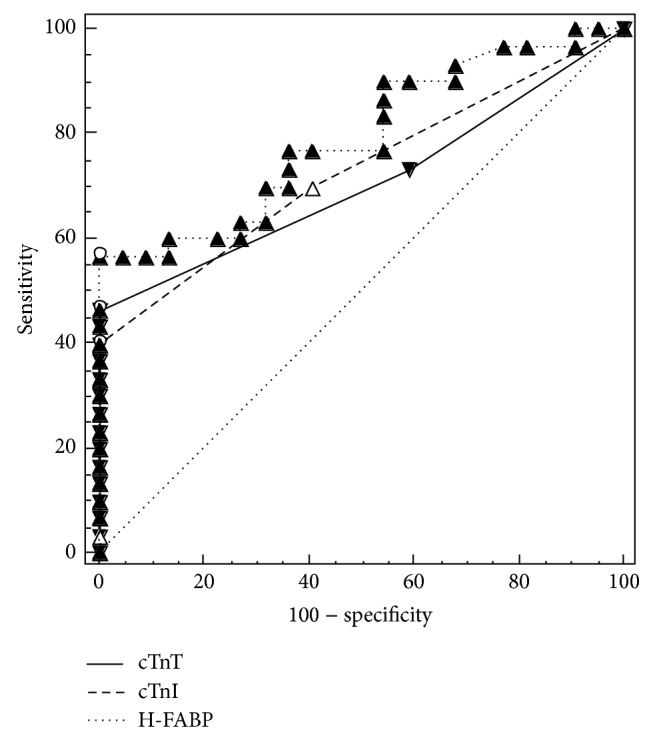
Diagnostic accuracy of cardiac biomarkers. Receiver operator characteristic (ROC) curves that are used to derive the cut-off concentrations for various cardiac markers [12]. See text for sensitivity and specificity values.

**Figure 3:**
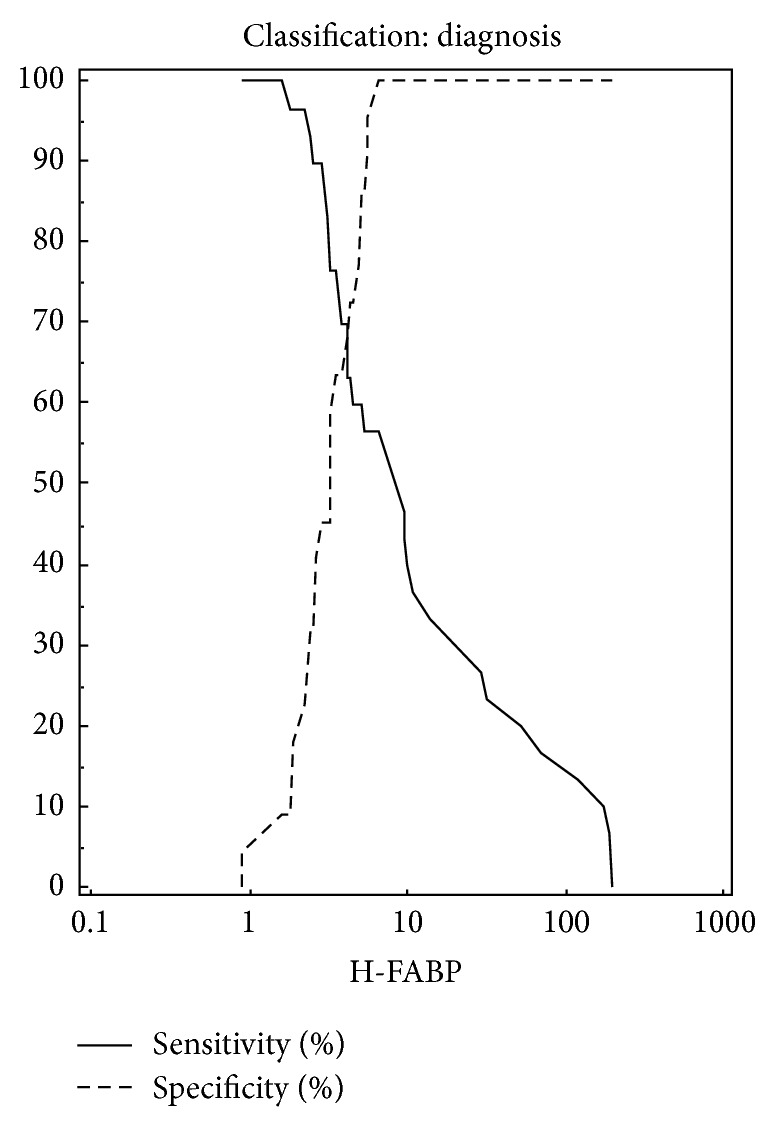
Plot versus criteria value for H-FABP. In this graph the sensitivity and specificity are plotted against the different criterion values.

Data used: pretest probability 35%, cases 30, and controls 22. The diagnostic accuracy for NSTE-ACS, as quantified using AUC, is shown in Figure 2. The AUC for H-FABP (0.79, 95% CI (0.66– 0.89))  was highest among the three. At 100% specificity and 100% positive predictive value, cTnI had the sensitivity of 40%; at the serum concentration level above 0.009 *μ*g/L, cTnT at the serum concentration level above 9 *μ*g/L; the sensitivity was 46.7%, specificity 100%, and positive predictive value of 100%. H-FABP at the serum concentration above 6.5 ng/mL, the sensitivity was 56.7%, specificity 100%, and positive predictive value of 100% (see Figure 3). At the optimal criteria for all the three biomarkers the negative predictive value was 55%, 58%, and 63%, respectively. The negative likelihood ratio was 0.6, 0.53, and 0.43, respectively. H-FABP had less false positives.

Table 4 displaying the estimated specificity for a range of fixed and prespecified sensitivities of 80, 90, 95 and 97.5% as well as estimated sensitivity for a range of fixed and prespecified specificities with the corresponding criterion values with the confidence intervals. This table can be used to calculate the positive predictive value and negative predictive value applicable in individual clinical settings when one knows the prior probability of disease (pretest probability or prevalence of disease) [13].

## 4. Discussion

Early diagnosis of AMI facilitates rapid and appropriate triage of patients within the Accident and Emergency Department, helping to prevent inadvertent discharge of patients with AMI. It also avoids delay in administering treatment for acute MI and reduces the possibility of patients without acute MI being given treatments from which they will not benefit, and which have the potential to cause significant harm. The working diagnosis of NSTE-ACS is a rule out diagnosis based on the ECG, that is, lack of persistent ST elevation. The 12-lead ECG is an important tool for early detection of acute MI, but it has significant limitations [14], and also the interpretation of the 12-lead ECG is dependent on the experience of the physician. Biomarkers (troponins) further distinguish NSTE-ACS and unstable angina. Diagnostic findings and risk stratification are closely linked [15]. In the clinical setting, a test with high ability to rule out (negative predictive value) and correctly diagnose ACS (positive predictive value) is of paramount interest. NACB recommends [16] for patients who present within 6 hrs. of the onset of symptoms (level of evidence: B (class ii b), an early marker of myocardial necrosis may be considered in addition to a cardiac troponin and had suggested that myoglobin is the most extensively studied marker for this purpose and secondly a rapid “rule in” protocol with frequent early sampling of markers of myocardial necrosis if tied to therapeutic strategies (level of evidence: C). A major drawback with cardiac troponins is that they are released relatively slowly from damaged myocytes [17] and also have the drawback of biphasic release after tissue injury as first small amounts of cytoplasmic troponin is released before cytoskeletal troponin is released [18]. Myoglobin is a smaller protein as compared to CK-MB and cTn and is released as early as 1-2 hours after symptom onset during AMI [19]. Myoglobin is no longer useful in the routine assessment of patients for possible acute myocardial infarction (AMI). A number of studies have shown that myoglobin lacks both sensitivity and specificity for the cardiac myocyte [20–23] Myoglobin is also nonspecific due to low levels in heart tissue and high levels in skeletal muscle tissue [24]. The role of myoglobin will be relegated to that of total CK (without MB), LDH, and SGOT that is a Class III recommendation.

H-FABP is a sensitive biomarker for myocardial infarction [7, 25, 26] and one study showed increased sensitivity of 20.6% over troponin at 3–6 hours following chest pain onset [27] similar to our study, where in H-FABP had higher sensitivity of 58% (95% CI 37.4–74.5) when compared with cTnI 40% (95% CI 23–59) and cTnT 47% (95% CI 28–66). This sensitivity may be explained by the high concentration of H-FABP in myocardium compared to other tissues; the stability and solubility of H-FABP; its low molecular weight; 15 kDa compared to 18, 80, and 37 kDa for MYO, CK-MB, and cTnT, respectively [28–30]; its rapid release into plasma after myocardial injury—60 minutes after an ischemic episode [24]; and its relative tissue specificity [31]. The effectiveness of using the combination of H-FABP with troponins to diagnose MI within 6 hours is well reported [9, 32, 33]. These features of H-FABP make it an excellent potential candidate for the detection of acute myocardial injury. Body et al. evaluated the ability of 8 biomarkers to rapidly exclude AMI at the point of presentation. In their 705 patients heart fatty acid binding protein (H-FABP) had an AUC of 0.86 (95% CI 0.82–0.90), which was significantly higher than any other biomarker including cTnI [34]. In our study the discriminatory power for H-FABP was higher as indicated by AUC 0.79 versus cTnI 0.73 and cTnT 0.71.

**Figure 4:**
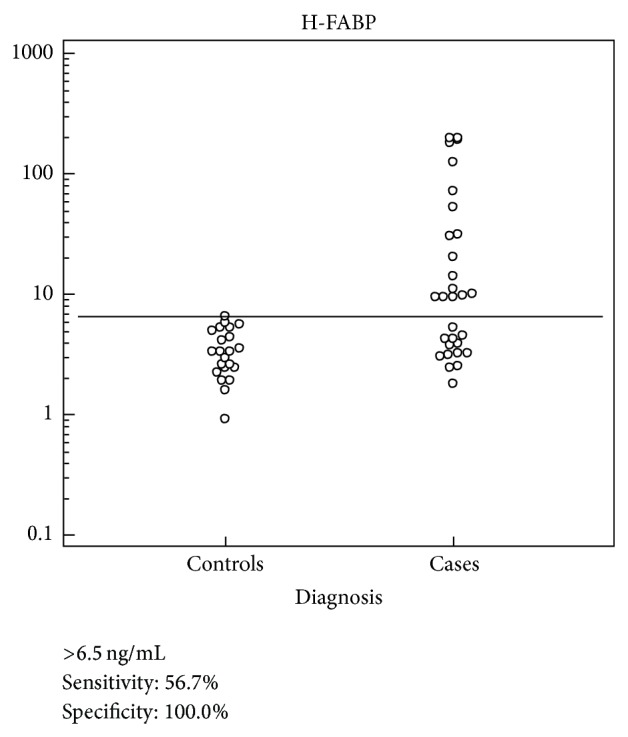
Dot diagram: the data of cases and controls are displayed as dots on two vertical axis. The horizontal line indicates the cut-off point with the best separation (minimal false-negative and false-positive results) between the two groups. 6.5 ng/mL as cut-off is very well in line with other studies [37–40]. The corresponding test characteristics sensitivity and specificity are shown below the display.

Xu et al. investigated the effectiveness of H-FABP for diagnosis of AMI in patients with different ethnic background and different time from symptom onset. Two hundred and eighty-nine patients admitted within 12 h after the onset of symptoms were recruited in the study. It gave the highest sensitivity (96% (95% CI: 91–98%)) and a comparable specificity (84% (95% CI: 76–89%)) to cTnI alone. The range of time point for our patients was 3.7–7.9 hours and 100% specific and 100%. PPV at the level of 6.5 ng/mL, the 63% NPV of H-FABP was higher than that of cTnI (55%) and of cTnT (58%) (see Table 3 and Figure 4). Therefore, the proportion of patients with a negative test resulting in correctly diagnosed by H-FABP is 10% and 5% more than cTnI and cTnT, respectively [35]. Hall et al. from their study concluded that more patients were diagnosed with NSTE-ACS and underwent coronary angiography after introducing the hs-cTnT assay. At the same time there was an increase in the frequency of coronary angiograms without signs of coronary artery disease (CAD) and fewer had significant dynamic cTnT concentration changes. So far no study has reported over diagnosis of NSTE-ACS with H-FABP [36].

The study of Hafidh Alhadi and Keith Fox highlights two important facts: first, H-FABP was more sensitive than myoglobin and second H-FABP had higher NPV than myoglobin. High sensitivity is essential for the early “rule in” of patients with AMI, and high NPV is important for the early “rule out” of AMI, since more than 90% of patients who present with acute chest pain to an Accident and Emergency Department do not have AMI [41].

In a study, out of the total number of patients who approached the Emergency Department with chest pain as the chief complaint, in the US, just 5 to 15% of them were found to be suffering from heart attacks or other cardiac diseases [42]. In countries like India, a sizable number of people seek emergency services with “Chest Pain” as the chief complaint. From the total sample of patients presenting with chest pain, only 5.5–8% were definitive cardiac ischemic events [43]. In a tertiary care institute like ours “rule out” is more important for effective patient treatment. In our study, H-FABP with 100% specificity has a higher sensitivity (56.7%) making it a better biomarker to rule out the disease. The positive likelihood ratio of H-FABP at 100% specificity (>6.5 ng/mL) is above 12.4, which is clinically very significant showing strong evidence for the presence of the disease. The prevalence rate of NSTE-ACS in a tertiary care hospital is higher than that of a general hospital; therefore, it becomes important to rule out the disease confidently. A high specificity test gives least false positives. The positive likelihood ratio of H-FABP (cut off 6.5 ng/mL, +LR 12.5, 95% CI: 1.8–86.8) is significantly higher than that of cTnI (cut off 0.009 *μ*g/L, +LR 1.71, 95% CI: 1.0–3.0) and cTnT (cut off 9.00 *μ*g/L, +LR 1.2, 95% CI: 0.8–1.9). So the level of 6.5 ng/mL of H-FABP has much better certainty about a positive diagnosis.

## 5. Conclusion

In our study we have shown that serum levels of H-FABP can be used in the early hours of chest pain to “rule out” the existence of NSTE-ACS in some and “rule in” in some and seems to be a preferred biomarker in the differential diagnosis of NSTE-ACS. We emphasize H-FABP testing as the main-line marker for triage of patients to assist in their optimal and efficient management. Though the number of cases is small, our observation might generate hypothesis for forthcoming studies.

## Figures and Tables

**Table 1:** Demographic variables of cases and controls.

Variable	Cases (*n* = 30)	Controls (*n* = 22)	*P*-value
Sex	M = 20, F = 10	M = 16, F = 6	
Age, yrs. (mean ± SD)	57.7 ± 11.1	45.3 ± 5.1	<0.001
Smoking *n* (%)	7 (23)	—	—
Alcohol *n* (%)	5 (17)	—	—
Hypertension *n* (%)	10 (33)	—	—
Diabetes *n* (%)	6 (20)	—	—
LAB. Parameters (mean ± SD)			
TC (mg/dL)	181 ± 53.68	179.5 ± 39.34	>0.05
LDL-C (mg/dL)	105 ± 48.58	107.3 ± 30.59	>0.05
VLDL-C (mg/dL)	32.60 ± 13.77	39.7 ± 16.54	>0.05
TG (mg/dL)	162 ± 70.15	198.4 ± 82.68	>0.05
HDL-C (mg/dL)	45.43 ± 11.36	32.5 ± 6.72	<0.001
TC/HDL-C	4.13 ± 1.19	5.6 ± 1.33	<0.001
Creatinine (mg/dL)	1.1 ± 0.2	1.015 ± 0.21	>0.05
Urea (mg/dL)	26.81 ± 10.1	23.6 ± 8.76	>0.05
CAG positive *n* (%)	23 (77)	—	—
ECG positive *n* (%)	26 (87)	—	—
2D-Echo positive *n* (%)	12 (40)	—	—

TC: total cholesterol, LDL-C: low density lipoprotein cholesterol, VLDL-C: very low density lipoprotein cholesterol, TG: triglycerides, HDL-C: high density lipoprotein cholesterol, CAG: coronary angiogram, ECG: electrocardiogram, and 2D Echo: two dimensional echocardiogram.

**Table 2:** Summary statistics of cardiac biomarkers in controls and cases.

Controls (*N* = 22)					
Variable	Mean ± SD	Median	5–95% CI		
cTnI (*μ*g/L)	0.01 ± 0.001	0.0	0.008–0.009		
cTnT (*μ*g/L)	8.6 ± 0.5	9.0	8.0–9.0		
H-FABP (ng/mL)	3.5 ± 1.5	3.3	1.32–6.02		

Cases (*N* = 30)					
Variable	Min.	Max.	25th Percentile	50th Percentile	75th Percentile

cTnI (*μ*g/L)	0	2	0.01	0.01	0.16
cTnT (*μ*g/L)	8	4700	8	9	175
H-FABP (ng/mL)	2	199	3.58	9.40	36.90

cTnT: serum cardiac troponin T, cTnI: serum cardiac troponin I, H-FABP: serum heart fatty acid binding protein, and CI: confidence interval.

**Table 3:** Receiver operating characteristics curve analysis.

Test characteristic	CTnI	cTnT	H-FABP
Criterion	>0.009 *μ*g/L	>9 *μ*g/L	>6.5 ng/mL
Area under the ROC curve (AUC)	0.73	0.71	0.79
95% Confidence interval	0.59 to 0.84	0.57 to 0.83	0.66 to 0.89
Significance level P (Area = 0.5)	0.0003	0.002	<0.0001
Sensitivity	40	46.7	56.7
95% CI	22.7–59.4	28.3–65.7	37.4–74.5
Specificity	100	100	100
95% CI	84.6–100.0	84.6–100.0	84.6–100.0
+LR	1.2	1.7	12.47
95% CI	0.8–1.9	1.0–3.0	1.8–86.8
−LR	0.6	0.53	0.43
95% CI	0.4–0.8	0.4–0.7	0.3–0.7
+PV	100	100	100
95% CI	73.5–100.0	76.8–100.0	79.4–100.0
−PV	55	57.9	62.9
95% CI	38.5–70.7	40.8–73.7	44.9–78.5

+PV: positive predictive value, −PV: negative predictive value, +LR: positive likelihood ratio, and −LR: negative likelihood ratio.

**Table 4:** Estimated specificity for a range of fixed and prespecified sensitivities and vice versa for cardiac biomarkers.

Variable	cTnI		
Estimated specificity at fixed sensitivity			
Sensitivity	Specificity	95% CI	Criterion (*μ*g/L)
80.00	59.09	31.82 to 72.73	>0.008
90.00	59.09	31.82 to 72.73	>0.008
95.00	59.09	31.82 to 72.73	>0.008
97.50	59.09	31.82 to 72.73	>0.008

Estimated sensitivity at fixed specificity			
Specificity	Sensitivity	95% CI	Criterion (*μ*g/L)

80.00	54.67	36.30 to 72.00	>0.0085
90.00	47.33	30.94 to 64.00	>0.0088
95.00	43.67	27.36 to 61.37	>0.0089
97.50	41.83	25.49 to 60.74	>0.0089

Variable	cTnT		
Estimated specificity at fixed sensitivity			
Sensitivity	Specificity	95% CI	Criterion (*μ*g/L)

80.00	40.91	10.47 to 59.09	>8
90.00	40.91	13.64 to 59.09	>8
95.00	40.91	13.64 to 59.09	>8
97.50	40.91	13.64 to 59.09	>8

Estimated sensitivity at fixed specificity			
Specificity	Sensitivity	95% CI	Criterion (*μ*g/L)

80.00	55.69	39.78 to 71.18	>8.6615
90.00	51.18	35.24 to 68.13	>8.8308
95.00	48.92	32.63 to 67.04	>8.9154
97.50	47.79	31.32 to 65.53	>8.9577

Variable	H-FABP		
Estimated specificity at fixed sensitivity			
Sensitivity	Specificity	95% CI	Criterion (ng/mL)

80.00	45.45	13.64 to 68.18	>3.15
90.00	45.45	20.26 to 77.27	>2.9
95.00	27.27	4.55 to 59.09	>2.3
97.50	9.09	0.00 to 26.18	>1.75

Estimated sensitivity at fixed specificity			
Specificity	Sensitivity	95% CI	Criterion (ng/mL)

80.00	60.00	36.67 to 76.67	>5.06
90.00	56.67	30.00 to 70.00	>5.46
95.00	56.67	33.33 to 70.00	>5.68
97.50	56.67	33.33 to 70.00	>6.06
